# Data on phytoplankton abundance in Malacca Straits, west coast of Peninsular Malaysia in August 2019

**DOI:** 10.1016/j.dib.2021.106893

**Published:** 2021-02-18

**Authors:** Erqa Shazira Sohaimi, Roswati Md Amin, Azwani Sahibu, Mohd Fadzil Mohd Akhir

**Affiliations:** a Faculty of Science and Marine Environment, Universiti Malaysia Terengganu, 21030 Kuala Nerus, Terengganu, Malaysia; b Institute of Oceanography and Environment, Universiti Malaysia Terengganu, 21030 Kuala Nerus, Terengganu, Malaysia

**Keywords:** Andaman Sea, Malaysia, Phytoplankton, Straits of Malacca, Southwest monsoon

## Abstract

In this article, the abundance of phytoplankton community structure in Malacca Straits (MS); from Port Klang to Langkawi Island are reported. The datasets include data from 25 selected sampling sites that were acquired in August 2019 on board the RV Discovery's cruise expedition. These data contain details on the density of phytoplankton (cell L^−1^), total number of species, volume seawater filtered (in L) and the concentration factors (ml) in MS. Data presented in this article consists of 163 species, including unidentified species from 6 phyla of phytoplankton, along with the percentage of a major community group in MS.

## Specifications Table

**Table:** 

Subject area	Biology
Specific subject area	Phytoplankton ecology
Type of data	Table Figure
How data were acquired	Inverted microscope (Leica DMIL) for phytoplankton count.
Data format	Raw Analysed
Parameters for data collection	Density of phytoplankton (cell L^−1^); Percentage of main phytoplankton group (%)
Description of data collection	Water samples were filtered through 20 µm of plankton net and immediately fixed in 5% formaldehyde (final concentration) for microscopic analyses using sedimentation technique.
Data source location	Malacca Straits (MS), West of Peninsular Malaysia; 25 stations from Middle MS (3°24′39.60″ N, 100°34′44.40″ E) to the Andaman Sea (6°8′2.36″ N, 99°1′45.34″ E).
Data accessibility	All data are provided in this article

## Value of the Data

• The samples were obtained during the Southwest Monsoon in the Malacca Straits (MS). The findings presented can then be correlated with the effect of seasonal changes on the distribution of phytoplankton.
• Data can be used as a reference for analyzing phytoplankton variability in the coastal area and continental shelf.
• A comparative study on the phytoplankton population in the Malacca Straits and the Andaman Sea can be applied from these datasets.

## 1 Data Description

**Fig. 1:**
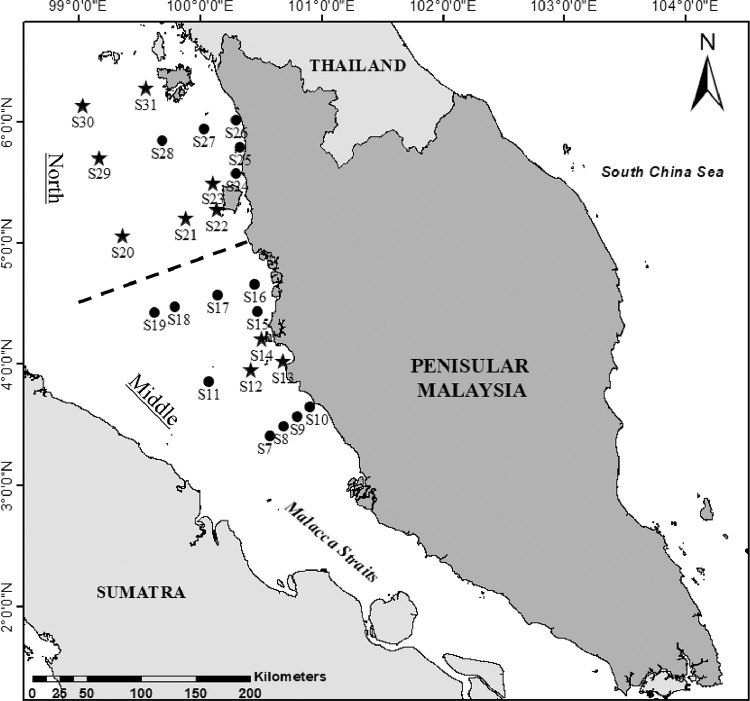
Map of sampling stations in North (Penang - Langkawi Island) and Middle (Port Klang - Matang) Malacca Straits of Peninsular Malaysia. Circle and star symbols indicate day and night time sampling, respectively.

The data given in this article provide details on the spatial variation of phytoplankton in Malacca Straits (MS) (Table 1 and Fig. 1). The datasets involve information on phytoplankton density consists of 163 species from 25 stations, including unidentified species. Data on phytoplankton density (cell L^−1^), total no. of species, volume seawater filtered (L) and the concentration factors (ml) in MS surface water are described in Table 2. The results on percentage abundance for the major group of phytoplankton in MS are presented in Table 3.

**Table 1:** Location of sampling stations.

Zone	Stations	Sampling date	Sampling Time	Longitude (E)	Latitude (N)	Depth (m)
Middle MS	S7	20/8/2019	09:15	100° 34′ 44.4′'	3° 24′ 39.6′'	57.7
	S8	20/8/2019	10:50	100° 41′ 16.8′'	3° 29′ 27.6′'	50.4
	S9	20/8/2019	12:00	100° 47′ 49.2′'	3° 34′ 15.6′'	38.3
	S10	20/8/2019	13:15	100° 54′ 21.6′'	3° 39′ 03.6′'	13.3
	S11	20/8/2019	18:15	100° 04′ 12.0′'	3° 51′ 21.6′'	64.6
	S12	20/8/2019	20:55	100° 25′ 01.2′'	3° 57′ 03.6′'	59.7
	S13	20/8/2019	23:26	100° 40′ 40.8′'	4° 01′ 22.8′'	8.4
	S14	21/8/2019	01:20	100° 30′ 01.7′'	4° 12′ 27.3′'	49.0
	S15	21/8/2019	07:00	100° 28′ 19.2′'	4° 25′ 58.8′'	7.0
	S16	21/8/2019	08:55	100° 26′ 42.0′'	4° 39′ 32.4′'	7.9
	S17	21/8/2019	11:00	100° 08′ 27.6′'	4° 34′ 30.0′'	54.6
	S18	21/8/2019	14:15	099° 47′ 35.6′'	4° 28′ 43.4′'	60.4
	S19	21/8/2019	16:15	099° 37′ 10.3′'	4° 25′ 50.7′'	53.7

North MS	S20	21/8/2019	22:00	099° 21′ 21.2′'	5° 03′ 28.6′'	68.2
	S21	22/8/2019	02:40	099° 52′ 37.1′'	5° 12′ 06.1′'	57.1
	S22	22/8/2019	04:40	100° 08′ 15.0′'	5° 16′ 24.9′'	26.7
	S23	22/8/2019	06:45	100° 06′ 00.7′'	5° 29′ 47.5′'	22.4
	S24	22/8/2019	08;20	100° 17′ 54.7′'	5° 34′ 34.8′'	6.0
	S25	22/8/2019	10:09	100° 19′ 28.1′'	5° 47′ 29.9′'	6.6
	S26	22/8/2019	11:44	100° 17′ 38.4′'	6° 00′ 59.3′'	7.3
	S27	22/8/2019	13:15	100° 02′ 00.5′'	5° 56′ 40.5′'	29.1
	S28	22/8/2019	15:20	099° 41′ 09.9′'	5° 50′ 55.5′'	53.9
	S29	22/8/2019	19:50	099° 09′ 54.1′'	5° 42′ 17.9′'	60.5
	S30	22/8/2019	23:18	099° 01′ 45.3′'	6° 8′ 2.364′'	70.6
	S31	23/8/2019	01:10	099° 33′ 01.2′'	6° 16′ 39.9′'	48.8

**Table 2:** Data on density of phytoplankton species (× 10^2^ cell L^−1^), total no. of species, volume seawater filtered (L), and concentration factor (ml). Note: (-) no organisms found are described.

	Stations																								
	Middle MS													North MS											
Species	S7	S8	S9	S10	S11	S12	S13	S14	S15	S16	S17	S18	S19	S20	S21	S22	S23	S24	S25	S26	S27	S28	S29	S30	S31
**Bacillariophyta**																									
*Bacillaria paxillifera*	0.22	1.56	–	–	–	0.97	–	–	–	4.27	–	–	–	–	–	–	–	2.10	1.98	–	–	–	–	–	–
*Bacillaria paradoxa*	–	–	–	–	–	–	–	–	–	3.56	–	–	–	–	–	–	2.03	1.05	–	–	–	–	–	–	–
*Cylindrotheca* sp*.*	2.08	0.39	0.28	2.12	0.75	–	–	–	–	9.60	–	0.14	–	0.09	–	–	–	9.68	0.79	–	–	0.14	0.18	0.11	–
*Nitzschia behrei*	–	–	–	0.53	0.75	–	–	–	–	–	0.15	–	–	0.23	0.07	–	–	0.21	–	0.59	–	–	–	0.27	0.13
*Nitzschia draveillensis*	–	–	–	–	–	–	–	–	–	–	–	–	–	–	–	–	–	–	0.40	–	–	–	–	–	–
*Nitzschia longissima*	–	–	–	–	–	–	0.48	–	–	2.13	–	–	–	–	–	–	–	–	0.40	–	–	–	0.05	–	–
*Nitzschia lorenziana*	–	–	–	–	–	–	–	–	2.07	1.07	–	–	–	–	–	–	–	–	–	–	–	–	–	–	–
*Nitzschia sigma*	–	–	–	–	–	–	–	–	1.04	–	–	–	–	–	–	–	0.51	0.63	–	–	–	–	–	–	–
*Nitzschia sigmoidea*	–	–	0.28	1.06	–	–	–	–	2.07	–	–	–	0.03	–	–	–	2.03	–	–	0.59	–	–	–	–	–
*Nitzschia* sp. 1	–	0.78	–	–	–	0.65	2.40	0.53	–	–	–	–	–	–	–	–	–	–	–	–	–	–	–	–	–
*Nitzschia* sp. 2	–	–	0.85	4.25	4.11	0.32	0.48	0.71	9.33	2.13	0.07	0.14	–	0.09	0.04	–	–	–	0.40	0.59	0.41	–	0.14	0.11	–
*Nitzschia* sp. 3	–	–	–	–	–	–	–	–	8.29	0.71	–	–	–	–	–	–	–	0.84	0.40	–	0.24	–	0.09	0.11	–
*Nitzschia* sp. 4	–	–	–	–	–	–	–	0.356	–	2.13	–	–	–	–	–	–	–	–	–	–	–	–	–	–	–
*Pseudo-nitzschia fraudulenta*	–	6.63	–	5.84	3.36	–	–	–	2.07	–	–	–	–	–	–	–	1.52	–	–	–	–	–	–	–	–
*Pseudo-nitzschia pungens*	0.66	3.51	1.13	3.19	8.96	1.08	6.72	1.07	7.25	–	0.44	–	–	–	–	–	4.56	–	–	2.35	–	–	–	–	–
*Pseudo-nitzschia seriata*	–	2.34	–	10.09	4.85	0.22	–	0.36	6.22	–	–	–	–	0.19	0.14	–	–	–	–	–	0.16	–	–	–	–
*Cymbella* sp.	–	–	–	–	0.37	–	–	–	–	–	–	–	–	–	–	–	–	–	–	–	–	–	–	–	–
*Diploneis* sp.	–	–	–	–	–	–	–	–	–	–	–	–	–	–	–	0.85	–	–	–	–	–	–	–	–	–
*Gyrosigma* sp.	–	–	–	2.12	–	–	–	–	–	1.42	0.15	0.04	0.03	–	–	–	–	–	–	–	–	–	0.09	–	–
*Haslea* sp. 1	–	–	1.13	–	–	0.11	2.40	–	–	1.07	–	–	0.10	–	–	–	–	–	–	0.59	–	–	0.09	–	0.06
*Haslea* sp. 2	–	–	–	3.19	0.37	–	–	0.18	–	–	–	–	–	0.19	–	–	–	–	–	–	–	–	–	–	–
*Navicula directa*	–	–	–	–	–	–	–	–	–	1.07	–	–	–	–	–	–	–	–	–	–	–	–	–	–	–
*Navicula s*p. 2	–	–	–	2.12	–	–	–	0.53	–	0.36	0.15	0.07	–	–	–	–	–	–	–	–	–	–	–	0.04	–
*Navicula* sp. 3	0.11	–	–	–	0.37	–	1.44	–	3.11	–	–	–	–	–	–	–	–	–	–	–	–	–	–	0.04	0.16
*Navicula* sp. 4	–	–	–	–	–	–	–	–	–	–	–	–	–	–	–	–	–	–	0.396	–	–	–	–	–	–
*Pinnularia* sp.	0.22	0.78	–	0.53	–	–	–	–	3.11	–	–	–	–	0.09	–	–	–	–	–	1.76	–	–	–	–	–
*Plagiotropis* sp.	–	–	–	0.53	–	0.11	0.48	–	3.11	0.36	–	–	–	–	–	–	–	–	–	–	–	–	–	–	–
*Pleurosigma cuspidatum*	–	–	–	–	–	–	–	–	8.29	–	–	–	–	–	–	–	–	1.26	–	1.76	–	–	–	–	–
*Pleurosigma elongatum*	–	–	–	1.59	–	–	0.96	–	–	2.13	0.15	–	–	–	–	–	–	0.84	–	2.35	–	0.02	–	–	–
*Pleurosigma strigosum*	–	–	–	0.53	0.75	–	4.32	–	–	6.76	0.22	–	–	–	–	–	–	–	–	–	–	0.05	0.05	0.08	–
*Pleurosigma* sp. 2	–	–	–	–	–	–	–	–	–	–	–	–	–	–	–	–	–	–	2.77	–	–	–	–	–	–
*Entomoneis* sp.	–	–	–	–	–	–	–	–	3.11	3.91	–	–	–	0.05	–	0.85	0.51	–	–	–	–	–	–	–	–
*Surirella fastuosa*	–	–	–	–	–	–	0.96	–	3.11	4.98	–	0.04	0.07	–	–	–	0.51	1.05	1.19	0.59	–	–	–	–	–
*Thalassionema frauenfeldii*	0.22	–	–	9.03	9.71	0.32	12.00	0.89	12.44	1.07	0.15	0.25	0.17	0.56	–	2.56	–	–	1.98	11.73	1.30	0.17	0.09	0.23	0.22
*Thalassionema nitzschioides*	1.53	–	–	4.25	3.36	0.43	11.52	0.89	4.15	9.96	0.44	–	0.28	–	–	–	–	1.68	–	1.17	–	0.10	–	0.11	0.13
*Thalassiothrix* sp.	1.31	3.12	1.99	13.80	5.97	1.52	4.80	1.78	11.40	0.36	0.30	–	–	0.42	0.11	–	–	4.84	0.79	–	0.81	0.38	0.51	0.54	0.98
*Amphora crassa*	–	–	0.57	–	–	–	–	–	–	1.07	–	–	–	–	–	–	–	–	–	–	–	–	–	–	–
*Amphora* sp. 1	–	1.17	–	–	–	–	1.44	0.18	–	–	–	–	–	–	–	–	–	–	–	–	–	–	–	–	–
*Amphora* sp. 2	0.22	–	–	16.46	1.49	0.22	3.36	–	–	–	0.22	–	–	–	–	0.85	1.52	0.63	–	0.59	–	0.05	–	0.08	0.06
*Licmophora* sp.	–	0.78	–	1.06	–	0.11	0.48	1.24	8.29	0.36	0.15	0.04	–	–	–	–	–	0.21	–	–	–	–	–	0.04	–
*Asterionellopsis* sp.	–	–	–	–	–	–	–	–	7.25	2.84	–	–	0.14	–	–	–	–	–	–	–	–	–	–	–	–
*Lampriscus shadboltianum*	–	–	–	–	–	–	10.08	–	–	–	1.63	–	–	–	–	–	–	–	–	–	–	–	–	–	–
*Bellerochea horologicalis*	–	–	–	–	–	–	–	0.71	–	–	–	–	–	–	–	–	–	–	–	–	–	–	–	–	–
*Climacodium* sp.	–	–	–	–	–	1.30	10.08	4.09	10.36	–	–	0.04	–	0.05	–	3.41	1.01	1.26	–	–	0.16	–	–	–	0.09
*Eucampia cornuta*	0.11	10.92	4.26	6.37	–	0.54	2.40	1.78	20.73	–	0.07	–	–	–	0.18	–	–	–	0.79	–	–	–	–	–	–
*Eucampia zodiacus*	0.33	7.02	5.39	4.25	–	–	2.40	1.42	16.58	0.71	0.07	–	–	–	–	–	–	–	–	–	–	–	–	–	–
*Skeletonema* sp.	13.47	7.41	1.99	18.05	32.11	–	1.44	7.64	18.66	–	0.37	0.14	–	–	0.11	–	–	–	–	–	–	–	–	0.23	–
*Helicotheca tamesis*	–	–	–	–	–	–	–	0.89	–	–	–	–	–	–	–	–	–	–	–	3.52	0.08	–	–	–	–
*Hemiaulus hauckii*	–	0.39	–	–	15.68	–	11.52	–	2.07	–	–	–	–	0.09	–	–	–	–	–	–	–	–	–	–	–
*Hemiaulus chinensis*	0.99	31.60	3.40	5.84	29.12	5.41	85.92	50.13	403.17	12.09	1.04	–	0.59	0.09	0.36	1.71	0.51	–	–	–	0.24	0.02	0.28	0.11	0.09
*Hemiaulus membranaceus*	0.33	8.19	–	–	–	0.87	3.84	12.98	65.29	–	–	0.29	–	–	–	–	–	1.05	1.19	3.52	–	0.12	0.09	0.15	0.13
*Cerataulina dentata*	–	–	–	–	–	0.32	–	0.89	12.44	0.71	0.22	–	–	–	–	16.21	–	0.42	0.40	5.87	–	–	–	–	–
*Cerataulina pelagica*	–	1.56	–	6.37	7.84	0.11	2.40	1.07	24.87	1.07	1.85	0.14	0.10	0.37	–	0.85	9.12	–	–	1.76	–	0.38	–	0.42	0.13
*Bacteriastrum delicatulum*	–	10.92	8.23	7.96	21.28	0.54	16.80	10.49	55.97	1.78	0.81	–	–	–	–	11.95	1.01	–	1.58	7.04	0.16	0.05	0.09	0.15	–
*Bacteriastrum hyalinum*	–	6.24	5.39	4.78	19.04	1.19	21.12	18.49	54.93	2.49	0.67	0.58	0.24	0.05	0.57	–	–	0.21	–	8.80	0.41	0.10	0.28	0.23	0.06
*Bacteriastrum elegans*	–	–	–	–	0.75	–	–	–	–	–	–	–	–	–	–	–	–	–	–	–	0.97	–	0.05	0.11	–
*Bacteriastrum furcatum*	–	1.56	–	4.78	29.49	0.11	3.36	9.24	24.87	1.07	0.89	2.79	0.45	1.59	0.97	4.27	0.51	0.42	0.79	13.49	3.24	0.19	0.60	0.61	0.19
*Bacteriastrum varians*	–	–	–	–	0.75	–	–	–	–	–	–	–	–	–	–	–	–	–	–	–	–	–	–	–	–
*Chaetoceros affinis*	3.94	–	–	–	4.85	–	0.96	–	–	–	–	–	–	–	–	–	–	–	–	–	–	–	–	–	–
*Chaetoceros atlanticus*	–	–	–	1.59	2.99	–	2.40	0.71	1.04	–	0.22	–	–	0.37	–	–	–	–	–	–	–	0.07	–	–	–
*Chaetoceros coarctatus*	0.44	3.51	9.65	1.06	2.99	0.22	–	–	9.33	2.13	–	0.22	–	–	0.14	–	–	–	–	–	0.89	–	0.23	0.11	0.32
*Chaetoceros compressus*	–	–	–	–	12.69	0.97	16.80	10.31	94.31	8.53	3.85	4.34	2.02	3.87	2.19	20.48	–	–	1.98	11.15	2.43	0.36	12.71	1.64	0.69
*Chaetoceros costatus*	3.39	29.26	–	27.08	23.52	–	1.92	20.80	67.37	–	4.30	–	1.11	–	–	17.92	2.03	–	3.96	–	1.14	–	–	0.08	–
*Chaetoceros curvisetus*	0.99	10.92	0.85	8.50	12.32	0.22	–	9.60	–	2.49	–	–	–	–	0.36	–	4.05	–	–	–	0.41	–	–	–	–
*Chaetoceros danicus*	0.22	5.46	1.42	9.56	3.73	1.19	–	6.40	13.47	3.20	0.22	0.18	–	0.65	0.25	–	–	–	0.40	–	0.97	0.05	0.60	0.08	0.13
*Chaetoceros decipiens*	–	11.31	–	14.34	13.81	2.27	7.68	13.33	69.44	8.89	1.78	0.62	0.94	1.68	0.97	6.83	4.05	0.84	5.93	8.21	2.76	0.22	3.56	0.15	0.60
*Chaetoceros didymus*	–	–	–	–	4.85	–	–	1.78	–	–	0.37	–	–	–	0.21	4.87	–	–	–	–	–	–	–	–	–
*Chaetoceros diversus*	0.88	–	–	–	2.99	–	3.36	1.24	–	–	–	–	–	–	–	–	–	–	–	1.17	1.14	0.05	–	–	0.19
*Chaetoceros lorenzianus*	0.33	11.70	1.70	15.40	11.57	1.08	–	5.16	31.09	2.49	1.19	0.14	0.21	0.05	–	2.56	–	0.42	1.58	8.21	1.70	0.12	0.14	0.23	0.28
*Chaetoceros peruvianus*	0.11	0.39	–	1.06	1.12	–	–	–	1.04	–	0.15	0.11	–	0.14	0.04	–	–	0.21	–	1.76	0.73	0.05	–	–	0.06
*Chaetoceros pseudocurvisetus*	–	43.30	8.23	36.10	17.92	2.81	38.88	36.44	404.21	3.91	0.59	–	–	–	–	–	–	–	–	–	–	–	–	–	0.16
*Leptocylindrus danicus*	1.42	3.51	–	5.31	5.97	1.30	2.88	0.89	7.25	–	–	–	–	0.09	–	0.85	–	0.21	0.79	1.17	–	–	–	0.04	–
*Odontella aurita*	0.33	0.39	–	–	–	0.11	1.44	–	3.11	1.78	–	–	–	–	–	–	–	–	–	–	–	–	–	–	–
*Trieres chinensis*	–	0.39	–	1.59	–	–	5.28	1.24	16.58	2.13	0.15	0.04	0.07	–	0.07	1.71	–	–	0.79	0.59	–	0.02	–	–	–
*Trieres mobiliensis*	0.22	0.39	–	1.06	0.37	–	1.92	0.89	48.71	0.36	–	–	–	–	–	–	0.51	0.63	–	–	–	–	–	–	–
*Ditylum brightwellii*	1.53	1.17	–	3.72	0.75	0.54	2.88	1.60	4.15	0.36	0.15	–	–	–	–	2.56	–	0.21	1.98	17.60	0.16	0.05	0.05	–	0.06
*Cyclotella* sp.	–	1.95	0.28	0.53	–	0.11	10.08	0.71	6.22	9.96	0.15	–	–	–	–	–	1.01	1.68	3.96	12.32	0.16	0.17	–	–	–
*Lauderia annulata*	–	32.38	13.62	13.27	4.48	2.92	5.28	3.73	34.20	2.49	2.30	0.18	–	–	0.14	8.53	9.63	–	6.33	32.27	1.14	0.17	–	–	–
*Thalassiosira eccentrica*	0.44	–	0.28	2.12	1.49	0.22	3.84	–	4.15	2.84	–	–	–	–	0.14	–	0.51	1.26	1.98	28.75	1.05	0.14	0.23	–	0.25
*Thalassiosira oestrupii*	1.42	1.56	0.28	9.03	4.48	1.08	9.12	1.07	9.33	3.20	0.44	0.11	0.24	0.14	0.14	9.39	5.07	2.52	15.43	62.77	1.70	0.46	0.28	0.19	0.35
*Thalassiosira punctigera*	0.11	1.17	–	1.06	–	–	2.40	0.18	2.07	0.36	–	0.04	–	–	–	15.36	9.12	6.73	20.96	0.59	0.32	–	0.09	–	–
*Thalassiosira* sp. 2	–	–	–	–	–	–	–	–	–	–	–	–	–	–	–	–	–	–	–	–	–	–	–	–	0.09
*Thalassiosira* sp. 3	0.55	–	–	–	–	–	–	–	–	–	–	–	–	–	–	–	–	–	–	–	–	–	–	–	–
*Thalassiosira* sp. 5	–	–	–	–	–	–	–	–	–	–	–	–	–	–	–	–	–	–	–	–	–	0.05	–	–	–
*Thalassiosira* sp. 6	–	–	–	–	–	–	–	–	–	–	–	–	–	–	–	–	3.04	1.26	0.40	29.92	–	–	0.05	–	–
*Thalassiosira* sp. 7	–	–	–	–	–	–	–	–	–	–	–	–	–	–	–	1440.40	113.49	5.47	5.14	51.04	–	–	–	–	–
*Asteromphalus flabellatus*	–	0.39	–	–	0.37	0.11	0.96	0.36	–	–	–	–	–	–	–	–	–	–	–	0.59	–	–	–	–	–
*Asteromphalus hookeri*	–	–	–	–	–	–	–	–	–	–	–	–	–	–	–	–	–	–	–	–	0.16	0.05	0.09	–	0.06
*Corethron hystrix*	0.22	1.56	–	–	–	–	1.44	–	–	0.36	0.07	–	–	0.05	–	–	–	0.42	–	2.35	–	–	–	–	–
*Coscinodiscus centralis*	0.22	–	–	–	–	–	–	–	–	–	–	–	–	–	–	–	–	–	–	–	–	0.07	–	–	–
*Coscinodiscus granii*	0.22	–	–	0.53	0.75	0.11	0.96	–	–	2.13	0.07	–	0.14	–	–	–	–	0.21	–	2.35	0.24	0.05	0.09	0.04	0.06
*Coscinodiscus marginatus*	–	–	–	–	–	–	1.44	–	1.04	–	–	–	–	–	–	–	–	–	0.40	2.35	0.32	0.10	–	–	–
*Coscinodiscus oculus-iridis*	–	–	–	–	–	–	0.48	–	–	–	–	–	–	–	–	–	–	0.21	0.40	2.35	0.16	0.02	–	–	–
*Coscinodiscus radiatus*	–	–	–	0.53	–	–	–	–	–	–	–	–	0.03	–	–	–	–	–	–	–	–	–	–	–	–
*Palmerina* sp.	–	–	1.13	–	–	0.11	0.96	–	–	–	–	0.04	–	–	–	–	–	–	–	–	–	–	–	–	–
*Actinoptychus senarius*	–	–	–	–	–	0.11	0.48	–	–	–	–	–	–	–	–	–	–	–	–	–	–	–	–	–	–
*Triceratium* sp.	–	–	–	–	–	–	–	0.18	4.15	0.36	0.07	–	–	–	–	–	–	–	–	–	–	–	–	–	–
*Proboscia alata*	–	0.39	–	–	1.49	0.32	0.96	–	–	0.36	0.52	0.04	0.17	0.23	0.50	1.71	3.04	0.42	0.40	8.21	0.41	0.07	0.18	0.08	0.06
*Dactyliosolen* sp.	0.99	17.95	8.23	–	–	–	–	2.49	16.58	0.36	–	–	–	–	0.29	–	–	–	–	–	0.57	–	–	–	0.09
*Guinardia delicatula*	0.55	25.36	8.51	27.08	23.52	2.71	5.76	1.07	22.80	0.36	0.59	0.04	–	–	0.72	19.63	7.09	2.10	2.37	14.67	0.81	–	–	0.38	–
*Guinardia flaccida*	0.33	6.63	0.85	2.12	1.12	0.43	1.92	1.96	15.55	–	0.30	0.04	–	–	0.11	0.85	–	–	3.96	9.97	0.41	0.07	0.14	–	0.03
*Guinardia striata*	1.31	33.16	12.77	18.05	14.19	4.55	12.48	3.56	18.66	0.71	0.74	0.25	0.07	0.37	0.21	4.27	0.51	0.42	–	–	0.08	0.12	0.60	0.19	0.06
*Guinardia* sp. 1	–	–	–	–	–	–	–	–	–	–	–	–	–	–	–	–	–	–	–	60.43	0.49	–	–	–	–
*Rhizosolenia bergonii*	–	–	–	–	–	0.11	–	–	–	–	–	–	–	–	–	–	1.01	–	–	3.52	0.16	0.07	0.23	0.08	0.22
*Rhizosolenia cochlea*	–	–	–	–	–	–	0.48	–	3.11	0.36	–	0.07	0.03	0.05	–	1.71	–	0.21	1.58	3.52	0.08	0.05	–	0.04	–
*Rhizosolenia imbricata*	0.44	3.51	5.11	2.12	17.55	1.52	1.44	1.07	22.80	2.13	0.59	0.22	0.10	0.05	–	7.68	2.03	–	2.77	2.35	0.24	0.07	0.37	0.04	0.06
*Rhizosolenia setigera*	0.11	–	–	1.06	–	–	0.48	–	3.11	–	0.15	0.11	–	0.09	–	–	–	–	–	–	–	–	–	–	–
*Rhizosolenia setigera f. pungens*	0.44	–	0.57	2.65	8.59	–	2.88	0.36	10.36	2.84	0.22	0.22	0.10	0.05	0.21	0.85	0.51	–	0.40	4.11	0.16	0.07	0.42	0.11	–
*Melosira* sp.	–	3.12	–	–	–	–	–	–	–	–	–	–	–	–	–	–	–	–	–	–	–	–	–	–	–
*Paralia sulcata*	–	–	1.42	–	–	–	4.800	–	12.44	4.98	0.44	–	0.31	–	–	–	–	–	–	14.67	0.41	0.05	–	0.11	0.13
**Chlorophyta**																									
*Pterosperma undulatum*	–	0.39	–	–	–	–	–	–	–	–	–	–	–	0.47	0.18	–	0.51	–	–	–	–	0.14	–	0.42	0.50
*Pterosperma* sp. 1	–	–	–	–	–	–	–	–	–	–	–	–	–	–	–	–	–	–	–	–	–	–	0.46	–	–
**Cyanobacteria**																									
*Trichodesmium* sp.	0.22	–	–	–	–	0.43	0.96	0.18	44.57	–	0.74	2.57	2.23	3.59	0.36	1.71	–	1.68	1.58	–	1.38	0.07	4.44	3.40	0.98
Cyanobacteria sp.	–	4.29	4.82	–	–	13.10	4.32	–	–	–	–	–	–	–	–	–	21.28	–	–	45.76	0.16	–	1.29	–	0.19
**Haptophyta**																									
*Phaeocystis* sp.	–	–	–	–	–	–	–	0.53	–	–	0.81	–	–	–	–	–	–	–	–	–	–	–	–	–	–
**Miozoa**																									
*Dinophysis caudata*	0.11	–	–	–	–	–	–	–	–	–	–	–	–	–	–	–	–	0.84	7.12	5.87	–	0.02	–	–	–
*Dinophysis nasuta*	–	–	–	–	–	–	–	–	–	–	–	–	–	–	–	–	0.51	–	–	–	–	–	0.18	0.04	0.09
*Dinophysis miles*	–	–	–	–	–	–	–	–	–	–	–	–	–	–	0.21	–	–	0.21	0.79	–	–	–	–	–	–
*Histioneis costata*	–	–	–	–	–	–	–	–	–	–	–	–	–	–	–	–	–	–	–	–	–	–	–	0.08	0.09
*Ornithocercus magnificus*	–	–	–	–	–	–	–	–	–	–	0.07	0.07	0.03	–	0.04	–	–	–	–	–	–	–	–	0.08	–
*Ornithocercus* sp. 1	–	–	–	–	–	–	–	–	–	–	–	–	–	–	–	–	–	–	–	–	–	–	0.14	–	0.03
*Phalacroma rotundatum*	–	–	–	–	–	–	–	–	–	–	–	–	–	–	–	–	–	–	–	–	–	–	–	0.08	0.03
*Tripos brevis*	–	–	–	–	–	–	–	–	–	–	–	–	–	–	0.04	–	–	–	–	1.17	–	0.02	–	–	–
*Tripos furca*	–	–	0.28	–	–	–	0.48	–	–	–	–	–	–	–	–	–	–	–	–	2.93	–	0.05	–	0.04	–
*Tripos fusus*	–	–	–	–	0.37	0.11	0.48	0.18	–	–	–	0.07	0.03	–	–	–	0.51	0.21	0.40	0.59	0.16	0.05	0.14	0.04	–
*Tripos macroceros*	–	–	–	–	–	–	–	–	–	–	–	–	–	0.05	–	–	0.51	–	0.40	–	–	0.02	0.05	–	–
*Tripos porrectus*	–	–	–	–	–	–	–	–	–	–	–	–	–	–	–	–	–	–	–	–	–	0.02	–	–	–
*Tripos trichoceros*	–	–	–	–	–	–	–	–	–	0.36	–	–	–	0.05	0.07	–	–	–	–	1.17	–	–	–	0.11	–
*Gonyaulax* sp.	–	–	–	–	–	–	–	–	–	–	0.44	0.11	0.17	0.33	–	–	–	–	0.40	1.17	0.32	0.46	0.14	–	0.13
*Alexandrium minutum*	–	–	–	–	–	–	–	–	–	–	–	–	–	–	–	–	–	–	–	–	–	–	–	–	–
*Pyrophacus horologium*	–	0.39	–	–	–	–	–	–	–	–	–	–	0.07	0.05	–	1.71	–	0.21	0.40	–	–	0.07	–	–	0.03
*Pyrophacus steinii*	–	–	–	–	–	–	–	–	–	–	–	0.04	–	0.05	0.04	–	–	–	–	–	–	–	–	–	–
*Pyrocystis* sp.	–	–	–	–	–	–	–	–	–	–	–	–	0.14	0.05	–	–	–	–	–	–	–	–	–	–	–
*Syltodinium undulans*	–	–	1.42	–	1.87	–	–	–	3.11	0.71	–	–	0.10	0.33	0.21	0.85	–	–	–	–	0.49	1.46	0.32	0.23	0.38
*Akashiwo* sp.	–	–	–	–	–	–	–	–	–	–	–	–	–	–	–	–	–	–	–	–	–	–	–	–	0.03
*Gyrodinium britannia*	–	–	–	–	–	–	–	–	–	–	0.37	–	–	–	–	–	–	–	–	–	–	–	–	–	–
*Gyrodinium* sp. 1	–	–	–	1.06	–	–	1.44	–	–	–	–	–	–	–	–	–	–	–	–	–	–	–	–	–	–
*Karenia* sp.	–	–	–	–	–	0.11	–	–	–	–	–	–	–	0.05	–	–	–	0.42	–	–	–	0.60	–	–	–
*Nematodinium armatum*	–	–	–	–	–	–	–	–	1.04	–	–	–	–	–	–	–	–	–	–	–	–	–	–	0.27	–
*Diplopsalopsis* sp.	–	–	–	–	–	–	–	–	–	–	–	–	–	–	–	–	–	–	–	–	0.24	0.26	0.37	0.27	0.16
*Blepharocysta splendor-maris*	–	0.39	–	0.53	0.37	–	–	–	–	–	–	0.14	–	–	–	–	–	0.42	–	–	–	–	–	–	–
*Podolampas* sp.	–	–	0.57	–	–	–	–	–	–	–	–	0.04	0.03	0.05	–	–	–	–	–	–	0.08	–	–	–	–
*Archaeperidinium minutum*	–	–	–	–	–	–	–	–	–	–	–	–	–	–	0.04	–	0.51	–	–	–	–	0.07	–	–	–
*Preperidinium meunieri*	–	–	0.28	–	0.37	–	–	–	–	–	–	–	–	–	–	–	–	–	–	–	–	–	–	–	–
*Protoperidinium brevipes*	–	–	–	–	–	–	–	–	–	–	–	–	0.07	–	–	–	–	–	–	–	–	–	–	–	–
*Protoperidinium cerasus*	–	–	–	–	–	0.11	–	–	–	–	–	–	–	–	–	–	–	–	–	–	–	–	0.05	–	–
*Protoperidinium claudicans*	–	–	–	–	–	–	–	–	–	–	–	0.07	–	–	–	–	–	–	–	–	–	–	–	–	0.03
*Protoperidinium conicum*	–	–	–	–	–	–	–	–	–	–	–	–	0.07	–	–	–	–	–	–	–	–	–	–	–	–
*Protoperidinium depressum*	–	–	–	0.53	–	–	–	–	–	–	–	–	0.07	–	–	–	0.51	–	–	0.59	–	–	–	0.04	–
*Protoperidinium divergens*	–	–	–	–	–	–	–	–	–	–	–	–	–	–	–	–	–	–	0.40	–	0.08	0.02	–	–	–
*Protoperidinium quarnerense*	0.11	0.39	–	–	0.37	–	–	–	–	–	0.07	–	–	–	–	1.71	–	0.21	–	–	0.16	–	–	–	–
*Protoperidinium steinii*	0.33	–	–	–	–	0.11	–	–	–	–	–	–	–	0.05	–	–	–	0.42	–	–	0.08	0.05	–	0.04	0.03
*Prorocentrum balticum*	–	–	0.28	1.59	1.49	0.11	–	–	–	–	0.15	–	0.10	0.09	0.04	–	1.52	–	–	1.17	–	–	0.23	0.15	0.06
*Prorocentrum micans*	–	–	–	–	–	–	–	–	–	–	–	–	–	–	–	–	–	–	–	–	–	0.12	–	–	0.09
*Prorocentrum gracile*	–	–	–	–	–	0.11	0.48	–	–	0.36	–	–	–	0.05	–	–	–	–	–	0.59	–	0.10	–	–	–
*Scrippsiella* sp.	–	–	0.28	1.06	–	0.22	–	–	–	–	–	–	–	–	–	–	–	–	–	–	0.16	0.98	0.32	–	–
Dinoflagellate sp.1	–	–	–	–	–	–	–	–	–	–	–	–	–	–	–	–	–	–	–	–	–	–	0.05	–	–
Dinoflagellate sp.3	–	–	–	–	–	0.54	–	–	–	–	–	–	–	–	–	–	–	–	–	–	–	0.10	–	–	–
Unarmored dinoflagellate sp. 1	–	–	–	–	–	–	–	–	–	–	–	0.36	0.31	0.23	0.21	–	–	3.16	3.16	5.28	0.81	0.38	0.32	0.50	1.14
Dinoflagelates cyst	0.55	–	0.28	3.19	2.61	1.19	1.92	–	8.29	1.42	0.81	0.54	0.49	0.75	0.25	2.56	7.60	3.16	3.56	5.28	1.22	0.62	0.79	0.92	1.26
**Ochrophyta**																									
*Dictyocha* sp.	–	–	–	–	–	0.22	–	–	–	–	–	0.14	0.31	0.23	0.04	–	–	–	0.79	–	–	0.22	0.37	–	0.25
*Chromulina* sp.	–	–	–	–	–	–	–	–	–	–	–	–	–	–	–	–	–	–	–	–	–	–	0.05	0.04	–
Unidentified sp. 1	–	–	–	0.53	–	–	–	–	–	–	–	–	–	–	–	–	–	–	–	–	–	–	–	–	–
**Total density of phytoplankton**	**42.24**	**363.60**	**118.03**	**355.74**	**409.16**	**57.92**	**390.71**	**256.53**	**1830.32**	**156.09**	**33.33**	**15.85**	**12.05**	**18.48**	**10.96**	**1619.59**	**225.46**	**64.79**	**119.06**	**539.14**	**34.96**	**10.54**	**32.45**	**14.10**	**11.93**
**Total no. of species**	**404**	**932**	**416**	**670**	**1096**	**535**	**814**	**1443**	**1766**	**439**	**450**	**438**	**346**	**396**	**306**	**1898**	**445**	**308**	**301**	**919**	**431**	**443**	**702**	**369**	**378**
**Volume of seawater filtered (L)**	**642.9**	**642.9**	**642.9**	**385.7**	**642.9**	**642.9**	**90.0**	**90.0**	**90.0**	**90.0**	**90.0**	**90.0**	**90.0**	**90.0**	**90.0**	**90.0**	**90.0**	**90.0**	**90.0**	**90.0**	**90.0**	**90.0**	**90.0**	**90.0**	**90.0**
**Concentration factor (ml)**	**176**	**209**	**228**	**256**	**200**	**174**	**54**	**40**	**53**	**40**	**50**	**37**	**47**	**42**	**31**	**48**	**57**	**71**	**89**	**66**	**73**	**54**	**52**	**86**	**71**

**Table 3:** Percentage abundance (%) of the main group of phytoplankton in MS. Note: Others group consists of phylum Chlorophyta, Haptophyta Ochrophyta, and Unidentified sp. 1.

	Stations																								
	Middle MS													North MS											
Group (%)	S7	S8	S9	S10	S11	S12	S13	S14	S15	S16	S17	S18	S19	S20	S21	S22	S23	S24	S25	S26	S27	S28	S29	S30	S31
Cyanobacteria	0.50	1.18	4.09	–	–	23.36	1.35	0.07	2.43	–	2.22	16.21	18.50	19.44	3.27	0.11	9.44	2.60	1.33	8.49	4.41	0.68	17.66	24.12	9.79
Diatoms	97.03	98.39	93.03	97.61	98.18	71.78	97.42	99.65	96.89	98.18	89.56	73.74	64.74	65.15	84.31	99.47	85.17	83.12	84.05	86.72	84.69	44.24	70.09	52.30	53.70
Dinoflagellates	2.48	0.32	2.88	2.24	1.82	4.49	1.23	0.07	0.68	1.82	5.78	9.13	14.16	11.62	10.46	0.42	5.17	14.29	13.95	4.79	10.90	51.69	9.54	20.33	30.16
Others	–	0.11	–	0.15	–	0.37	–	0.21	–	–	2.44	0.91	2.60	3.79	1.96	–	0.22	–	0.66	–	–	3.39	2.71	3.25	6.35

## 2 Experimental Design, Materials and Methods

### 2.1 Sampling

All data were collected in Malacca Straits (MS) during a scientific expedition of MS on 20th – 23rd August 2019 onborad the UMT's RV Discovery. The sampling stations (S7-S31) were associated with the sampling site during a scientific expedition to Langkawi International Maritime and Aerospace exhibition (LIMA’19) in March 2019. Two areas were split between the sampling sites: Middle MS (S7-S19; Port Klang - Matang) and North MS (S20-S31; Penang - Langkawi Island). Surface water samples (∼1 m) were obtained using water pump and the sample were then filtered through 200 μm and 20 μm of serial plankton net; maintaining in a bucket filled with seawater, to minimize potential damage and clogging. Both samples were stored in one bottle sample and immediately preserved with 5% of formaldehyde at final concentration.

### 2.2 Species identification and phytoplankton cells counting

The identification of species was based on morphological characteristics according to the reference of phytoplankton identification [1], [2], [3], [4] and verified with AlgaeBase (https://www.algaebase.org). Species identification of phytoplankton cells was observed under an inverted microscope Leica DMIL at 200 × and 400 × magnification using sedimentation technique (Utermöhl method) [5]. Samples were concentrated first overnight with a ratio of 1:25 to 1:100 of dilution depends on the concentration of the samples. A total of a minimum of 300 cell units were counted in order to maintain an acceptable accuracy for the whole sample [6]. The samples were determined as far as possible at species level.

## CRediT Author Statement

**Erqa Shazira Sohaimi:** Conceptualization, Resources, Visualization, Writing – Original Draft; **Roswati Md Amin:** Conceptualization, Visualization, Writing – Review and Editing, Supervision, Funding acquisition, Project Administration; **Azwani Sahibu:** Resources; **Mohd Fadzil Mohd Akhir:** Funding acquisition, Project Administration.

## Ethics Statement

The authors declare this content is the author's original work, which has not been previously written elsewhere.

## Declaration of Competing Interest

The authors announce that they do not have any competing financial interests or personal relationships that could have an effect on the work stated in this paper.
